# Modern contraceptive methods utilization and associated factors among reproductive aged women in rural Dembia District, northwest Ethiopia: Community based cross-sectional study

**Published:** 2017-06

**Authors:** Shibihon Debebe, Miteku Andualem Limenih, Belete Biadgo

**Affiliations:** 1 *University of Gondar Hospital, Laboratory, Gondar, Ethiopia.*; 2 *University of Gondar, College of Medicine and Health Sciences, Department of Midwifery, Gondar, Ethiopia.*; 3 *University of Gondar, College of Medicine and Health sciences, School of Biomedical and Laboratory sciences, Department of Clinical Chemistry, Gondar, Ethiopia.*; Shibihon Debebe and Miteku Andualem Limenih are both co-first author.

**Keywords:** Contraceptive utilization, Ethiopia, Modern contraceptive

## Abstract

**Background:**

Improving women’s health through modern contraceptive methods utilization is the key strategy to prevent unwanted pregnancy and its complication. However, there was limited evidence on utilization of modern contraceptive methods in the study area.

**Objective:**

This study identified factors affecting utilization of modern contraceptive methods among women at reproductive age group in rural areas of Dembia district, 2015.

**Materials and Methods:**

Community based cross sectional study was conducted in 2015 in Dembia District. Multi-stage sampling technique was used to select a total of 616 study participants. Bivariate and multivariable logistic regression model was fitted to identify factors associated with modern contraceptive utilization. Odds ratio with 95% confidence interval (CI) was computed to determine the level of significance.

**Results:**

Modern contraceptive methods utilization was found to be 31.7% 95%CI (28.0-35.3). Age [Adjusted odds ratio (AOR): 1.94, (95%CI: 1.170-3.216)]**,** women who has educated husband [AOR: 0.28, (95%CI: 0.117-0.666)], Marital status [AOR: 2.81, (95%CI: 1.344-5.855)] and Spousal announcement about family planning issues [AOR: 2.58, (95%CI: (1.276-5.202)] were factors associated with modern contraceptive methods utilization.

**Conclusion:**

Modern contraceptive methods utilization was found to be low. Providing educational opportunities, creating awareness about contraception and effective counseling would increase modern contraceptive methods utilization.

## Introduction

Family planning (FP) is the decision of couples or individuals to have the required number of children at the appropriate time through utilization of contraceptive methods for the purpose of delaying, spacing or limiting (1). FP inadequacy, in developing countries, contributed significantly to poverty and ill health (2). Around $5.7 billion could be saved by avoiding unintended pregnancies and unsafe abortions through FP (3). Current projections show a persistent increase in population with the global population estimated to reach between 7.5 and 10.5 billion by 2050 (4). Africa’s population is projected to account for 21% of the world population by 2050 up from just 9% in 1950 (5). Ethiopia with a population of nearly 87 million in 2014 is the 2^nd^ most populous nation in sub-Saharan Africa, with a continuing fast growing population of 2.6% per year (6). The maternal mortality ratio (MMR) is 420 per 100 000 live birth women aged 15-49, with an estimated 32% of all maternal deaths attributed to unsafe abortions secondary to unintended pregnancy (7). 

The government of Ethiopia implemented an explicit population policy in 1993 with an overall objective of coordinating the country’s population growth rate with that of the economy. One of the major strategies of the policy has been to increase FP program and rises contraceptive prevalence to 55% by 2020 (8). The widespread implementation of FP represents one of the most extreme changes of the 20^th^ century. The increasing use of contraception around the world has given couples the aptitude to choose the number and spacing of their children and has had great lifesaving benefits (9). 

FP is one of the most successful development interventions of the past 50 years which is unique in its range of potential benefits, encompassing economic development, maternal and child health, educational advances, and women’s empowerment (3). Increment of contraceptive methods utilization can significantly reduce the costs of achieving selected Sustainable development goals and directly subsidize to reductions in maternal and child mortality (10). 

FP coverage varies considerably across regions, with about 3% of women in the Somali region reporting using modern contraception compared to about 60% in Addis Ababa and urban women are five times more likely to use contraceptives than rural women (11). Even though there is an increment of modern contraceptive methods utilization from time to time, currently 24% of matrimonial women either do not need any more children or they need to wait for two or more years before having another child, they were not using any form of contraception (12). Globally, contraceptive methods utilization helps to prevent a predictable 2.7 million infant deaths and the loss of 60 million healthy lives in a year (13). Even though these much benefits of contraceptive methods utilization are known, at present around 222 million women have insufficient access to contraceptives (12). 

In Ethiopia, the total fertility rate in rural areas (5.5 children per woman) is higher than urban area (2.6 children per woman) by almost 3 children per woman (11). If all unmet need for modern contraceptive methods were satisfied, maternal mortality would drop by almost 1/3^rd^ from current levels, and unplanned births and unsafe abortions would decline by 89-92% (8). In the previous few decades, FP programs have played a major part in raising the prevalence of contraceptive utilization from less than 10-60% and reducing fertility in developing countries from 6 to 3 births per woman (14). Although the overall contraceptive prevalence has been increased, the use of modern contraceptive method differs significantly among regions, urban and rural areas (15). 

Therefore, there was limited evidence on modern contraceptive methods utilization in rural areas of Dembia district. The purpose of this study was to assess utilization of modern contraceptive methods and associated factors among women of reproductive age group in rural kebeles of Dembia district.

## Materials and methods

A community based cross-sectional study was conducted from 1^st^-30^th^ June 2015 in Dembia district. Koladiba is the town of the district. According to information gained from the district health office, the woreda has 45 kebeles (40 peasant association and 5 urban kebeles). There are 10 health centers and 45 health posts in the district that are owned by the government for maternal health care service provisions including FP. 

**Inclusion and exclusion criteria**

All women who are at reproductive age group (15-49 yr) in the selected kebeles and who lived in the study area for at least six months were included whereas those women who were severely ill and unable to communicate during the study period were excluded from the study. 

**Sample size determination**

The sample size was determined using single population proportion formula with the assumption of 95% confidence interval (CI), a margin of error of 5% and taking 22.5% contraceptive prevalence of Ethiopian Demographic and Health Survey (EDHS) 2011 and a design effect of 2. To avoid the effect of the design that decreases the representativeness of the study, we used design effect. To compensate the non-response rate, 10% of the determined sample was added up to the calculated sample size and the final sample size was 616.

**Sampling techniques**

Multi-stage sampling technique was used. From 40 rural kebeles of the woreda 5 were designated by simple random sampling method. In the second stage sample size was dispersed to each kebeles rendering to chances proportional to size of each kebele and households were selected by systematic random sampling technique, the kebele office was considered a frame of reference and a pen was rotated and the first household was selected where the tip of the pen pointed and the interviewers interview after obtaining a verbal consent from subjects from each household in every 4th house. Sample distribution based on the selected kebeles was 103 (16.7%, 141 (22.9%), 173 (28.1%), 120 (19.5%) and 78 (12.5) households for Tezeba, Sanikisa, Abirijiha, Jenda and Janigua respectively. Finally, women of reproductive age who full fill the eligibility criteria in the selected households were requested to participate in the study. When two or more women were in a family, only one of them was asked using lottery method. 

**Data collection procedure and quality assurance**

A pre-tested structured questionnaire was used. The English language questionnaire was translated into the local language of Amharic, and then decoded back to English by other people who are skillful in both languages to keep the consistency of the questionnaires. Ten grade 10 completed girls ordered the structured questionnaire, after a 2-day training session that encompassed evidence about the relevance of the study, privacy of information, study participants' rights, informed consent, interview techniques, and hands-on demonstration of the interview. Four diploma holder clinical nurses controlled the data collection procedures. Observation involved revising all questionnaires on daily basis, followed by meetings with the data collectors to confer on any problems faced during data collection. At last data cleaning and editing were completed.

**Ethical consideration**

Ethical clearance was obtained from the research and ethics committee of Bahir Dar University. Further permission was obtained from the woreda Health Office, and heads of kebele administrations. The participants were given written consent forms as well as verbally informed about the purpose of the study and personal identifiers were not included in the written questionnaires to ensure participants` confidentiality. 

**Statistical analysis**

The Data were entered into a computer using Epidemiological Information (EPI-INFO) version 6 software and exported to SPSS (Statistical packages for social science) version 20. Data were analyzed using descriptive statistics, binary and multiple logistic regression analyses were used to identify associated factors. Variables having P≤0.2 in the bivariate analysis were fitted into multiple logistic regression model to control the effect of confounding. Crude and adjusted odds ratio with their 95% CI were calculated to determine the strength and presence of association. P≤0.05 was considered to declare the level of significance. Recent FP methods utilization has reserved a value of 1 if the respondents stated recently using and zero else. 

The reference group of each independent variable had a value of 1, and the value for other groups was compared to that of the reference category. Odds ratio (OR) <1 infers that persons in that category have a lower likelihood of utilization of FP service than persons in the reference category. Similarly, OR >1 was designated increase probability of reporting current utilization of FP services.

## Results

**Socio-demographic characteristics**

A total of 616 women included in the study, yielding a response rate of 99.8% with non-response rate of 0.2% and the mean age of the respondents was 29.9±7.2 yr. Regarding to the age distribution, it was realized that about (29%) of them were youth (15 to 24). The majority of the participants (77.8%) and their husband (60.3%) were unable to read and write. Four hundred seventy-nine (77.9%) were married, almost all (99.5%) were Orthodox Christians and 495(80.5%) women were housewives. A large proportion of the women had more than four children 302 (49.1%) and 152 (24.7%) had 2 to 3 children (Table I).

**Information and utilization of modern contraceptive methods**

Five hundred eight (82.6%) had heard about modern contraceptives and the major sources of information were government health institutions 385 (75.8%). From those they had heard about modern contraceptives, over 3/4^th^ of the respondents (78.1%) knew two and above methods, 62 (12.2%) injectable only, 49 (9.6%) pills only. Less than half of the women described debating the issue about the use of contraceptives with their spouses 256 (41.6%). Of these, almost all 238 (93.0%) of the women approved of modern contraceptive methods with their husbands. Nearly half 274 (44.6%) in this study had always used one of the modern contraceptives. From those ever used women, Injectable surpassed the list of ever used (four into five women) 232 (84.7%), pills and implants were the least ever used 31 (11.3%) and 11 (4.0%) respectively. Want to have many children 240 (70.4%) was the major reason not ever used any modern contraceptive methods. Modern contraceptive methods utilization was 31.7%, 95%CI (28.0-35.3).Methods mostly used were injectable, pills and implants 151 (77.4%), 24 (12.3%) and 20 (10.3%) respectively (Table II). 

**Reasons given by women for lack of utilization of modern contraceptive methods**

**Figure 1:**
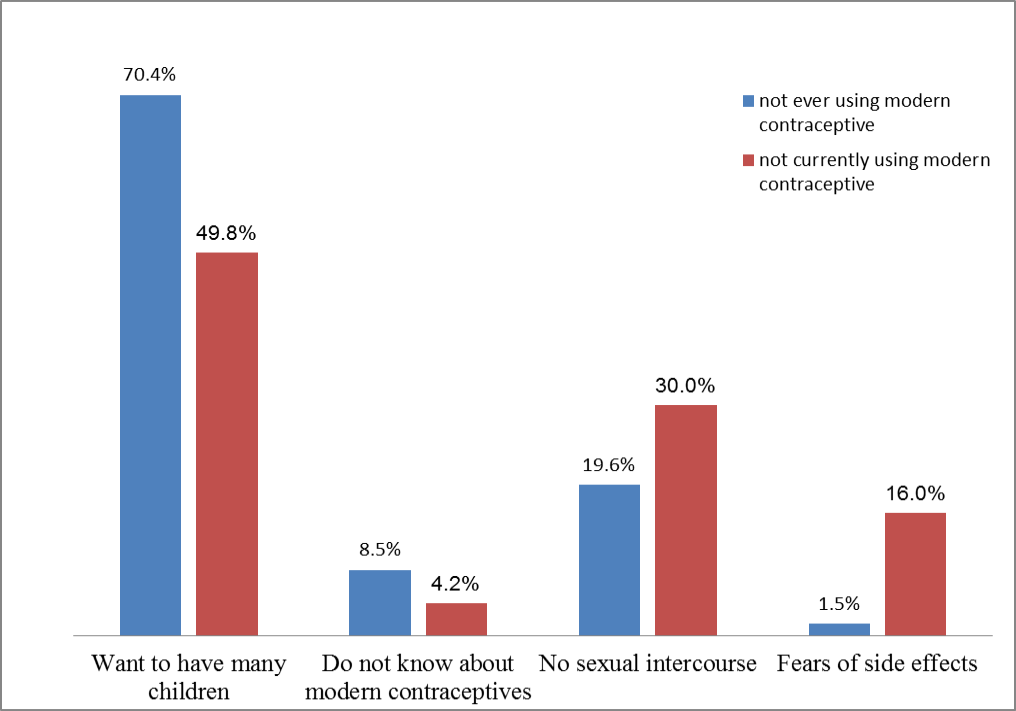
Reasons for not ever using and not currently using modern contraceptives among reproductive age women (15-49 years old) in rural kebeles of Dembia district, Ethiopia, 2015 (N=615

Of the 420 women who were not recently using any of modern contraceptive methods, almost all of them 415 (98.8%) need to use in the future. The most noticeable reasons for not using contraceptives were want to have many children 209 (49.8%), no intended to have sexual intercourse 124 (30.0%) and fears of side effects 67 (16%) (Figure 1).

**Factors associated with modern contraceptive methods utilization among reproductive age women in rural kebeles of Dembia district, Ethiopia**

Age [AOR: 1.94, (95%CI: 1.170-3.216)], women who has educated husband [AOR: 0.28, (95%CI: 0.117-0.666)], marital status [AOR: 2.81, (95%CI: 1.344-5.855)] and spousal announcement about FP issues [AOR: 2.58, (95%CI: (1.276-5.202)] were found to be significantly associated with modern contraceptive methods utilization in multivariate logistic regression analysis.

Those women who are 15-24 years old were 1.84 times more likely to use contraceptives compared to 35-49 yr old. The study participants who had 25-34 yr old were 1.94 times more likely to use contraceptive compared to 35-49 yr old. The more women’s husband educated, the more likely to use modern contraceptives. Those women who have husbands who cannot read and write were 0.20 times less likely to use contraceptives than those who have grade 7 and above. Those women whose husbands were in grade 1-6 were 0.28 times less probable to use contraceptives than those who were in grade 7 and above. 

The probability of using modern contraceptive was high among women who had noticed with their husbands. Those women who had noticed with their husbands were 2.58 times more likely to use modern contraceptive methods than those who did not. Being conjugal women were highly probable to use modern contraceptive methods compared to unmarried (widowed, singled and divorced). Women who have husband were 2.81 times more likely to use modern contraceptives than those who did not have husband (Table III).

**Table I.** Socio demographic characteristics of women at reproductive age group in Dembia district, Ethiopia, 2015 (N=615

**Variables**		**Number % (N=615)**
Age in year		
	15-24	178(28.9)
	25-34	267(43.4)
	35-49	170(27.7)
Education status of the women		
	Cannot read and write	478(77.7)
	Grade 1-6	73(11.9)
	Grade 7and above	64(12.4)
Education of husband		
	Cannot read and write	371(60.3)
	Grade 1-6	180(29.3)
	Grade 7 and above	64(10.4)
Religion		
	Orthodox Christian	612(99.5)
	Muslim	3(0.5)
Marital status		
	Married	479(77.9)
	Singled	35(5.7)
	Divorced	75(12.2)
	Widowed	26(4.2)
Living children in number		
	One	98(15.9)
	Two to three	152(24.7)
	Four and above	302(49.1)
	No living children	63(10.3)
Occupation of women		
	Housewives	495(80.5)
	Local drink makers	44(7.2)
	Petty merchant	36(5.9)
	Farming	40(6.4)

**Table II:** Information and practice of modern contraceptive methods distribution among women of age 15-49 years old in Dembia district, Ethiopia 2015 (N=615

**Variables**		**Number % **
Had information about contraceptives		
	Yes	508(82.6)
	No	107(17.4)
Source of information (n=508)		
	Government health intuitions	385(75.8)
	Community based reproductive health agents	17(3.3)
	Mass media	23(4.5)
	Neighbors	63(12.4)
	School	20(4.0)
Methods known (n=508)		
	Pills only	49(9.7)
	Injectable only	62(12.2)
	Two & above methods	397(78.1)
Spousal communication		
	Yes	256(41.6)
	No	359(58.4)
Spousal approval (n=256)		
	Yes	238(93.0)
	No	18(7.0)
Ever used of modern contraceptives (n=615)		
	Yes	274(44.6)
	No	341(55.4)
Methods ever used (n=274)		
	Pills	31(11.3)
	Injectable	232(84.7)
	Implant	11(4.0)
Currently using contraceptives		
	Yes	195(31.7)
	No	420(68.3)
Methods currently using (n=195)		
	Pills	24(12.3)
	Injectable	151(77.4)
	Implants	20(10.3)
Future intention to use contraceptives (n=420)		
	Yes	415(98.8)
	No	5(1.2)

**Table III:** Bivariate and multivariate logistic regression analysis on factors associated with current utilization of modern contraceptive methods among reproductive age women in rural kebeles of Dembia district, Ethiopia, 2015

**Variables**		**Modern contraceptive use**		**COR (95% CI)**	**AOR (95% CI)**	**P- value**
		**Yes**	**No**			
**Age in year**						
	15-24	65	113	2.48(1.518-4.053) *	1.84(1.058-3.213) *	0.03
	25-34	106	161	2.84(1.799-4.480) *	1.94(1.170-3.216) *	0.031
	35-49	32	138	1.0	1.0	0.010
**Husband education**						
	Can’t read and write	95	278	0.41(0.281-1.158)	0.2(0.085-0.455) *	≤0.01
	Grade 1-6	80	100	0.97(0.601-2.554)	0.28(0.117-0.666) *	≤0.01
	Grade 7and above	28	34	1.0	1.0	0.004
**Marital status**						
	Married	188	291	5.88(2.711-11.532) *	2.81(1.344-5.855) *	<0.01
	Singled	5	30	1.52(0.119-2.228)	0.40(0.102-1.588)	0.006
	Widowed/divorced	10	91	1.0	1.0	0.186
**Occupation of women**						
	Housewife	166	329	1.24(1.006-5.112) *	1.71(0.812-3.613)	0.151
	Local drink maker	15	29	1.27(1.070-8.584) *	2.80(0.981-8.008)	0.157
	Others	22	54	1.0	1.0	0.054
**Communication**						
	Yes	142	114	6.09(3.205-8.017) *	2.58(1.276-5.202) *	0.008
	No	61	298	1.0	1.0	
**Approval**						
	Yes	134	104	1.90(0.915-3.953)	1.82(1.276-5.202)	0.091
	No	69	308	1.0	1.0	
**Information have**						
	Yes	184	324	2.63(1.463-4.739) *	1.545(0.860-2.775)	0.145
	No	19	88	1.0	1.0	

* statistically significant associations. 1.0= reference category

## Discussion

The current study revealed that modern contraceptive methods utilization was 31.7%. This finding was higher as compared to a study conducted in Tanzania (12.5%), Congo (7.8%), Gondar town surrounding peasant association (11.0%), and Nigeria (5.2%) respectively (16-19). The possible reason might be increasing access to modern contraceptive service due to information delivery by health extension workers, different Non-Governmental Organizations (NGO's) and the presence of different mass media in the area by increment of government and private health institutions from time to time including health posts. 

However, this result was lower than Mojo town (38.3%) in Southern Ethiopia, Hosanna (67.4%) and Bangladesh (20-22). This difference might be due to variation in awareness of the people, availability of the contraceptive methods and the difference in study settings to access the service. 

The majority, 508 (82.6%) of the respondents had evidence about modern contraceptives methods. The major source of evidence was government health facilities 385 (75.8%). This result is in agreement with a study done in Northwest Ethiopia (93.1%), a study conducted in Osun state, Nigeria (82.4%), and a study in Mekele, Ethiopia (83%) (18, 23, 24). This might be due to advocacy and comprehensive community mobilization for modern FP at the respective area, due to increasing access to contraceptives, expansion of modern contraceptive methods by health workers, accessibility of a range of method choice and facilities given the service and due to the presence of high governmental focus.

In this study, two and more than two methods were known by 397 (77.5%) respondents. However, a study conducted in Tanzania rural district revealed that pill was the most known method (81.2%) and injectable was the second known (76.8%) and the finding of the study conducted in Shireendeselase, Ethiopia showed the similar result (19, 25). This showed that many modern contraceptive methods were becoming popular. The possible reasons for this might be, women are usually addressed by health education by rural health extension workers.

This study showed that only 3 methods were found to be used by respondents. These are injectable 151(77.4%), pills 24(12.3%) and implant 20(10.3%). Despite its low usage, it is encouraging that implants used as an contraceptive method by rural women since it helps to space for longer periods. Even though the respondents know more than 3 methods, most women used the injectable contraceptive. The finding of this study showed that none of the study participants mentioned male and female sterilization, IUCD and condom as contraceptive methods to space pregnancy or limit their number of children. These findings were in line with a study conducted in street women in northwest Ethiopia (24). The possible reason might be probably due to cultural influence, poor attitude towards the methods, shortage of awareness about these methods and lack of access.

In this study, the age of women and modern contraceptive methods utilization were strongly associated. As women`s age increases from 15 to 34 ages old, the possibility of using contraceptive methods was increasing. This finding was similar to the result of the study conducted in Nigeria, South Ethiopia, Mali, Congo and Bangladesh (16, 18, 21, 26, 27) respectively. The possible explanation might be in rural areas, this age group is the time at which most women engaged in different activities to fulfill their household needs and as a result, they want to space their birth. So, they prefer to utilize contraceptive methods. The other possible explanation might be due to increased experience sharing from peers, neighbors and this age group nowadays have women forum association to discuss the issue. This might increase their utilization rate. However, these findings were inconsistent with a study conducted in Mojo town and a study done in Kerman, Iran which showed modern contraceptive users showed a lower mean age than nonusers(20, 28). The possible explanation might be due to time variation and different socio-demographic events. 

This study showed that Women’s who have educated husband were more likely tend to utilize contraceptive methods. This finding has similar with a study finding in Hosanna, Southern Ethiopia (22). The possible explanation might be; education can improve awareness, can induce sharing of decision making power and can improve the attitudes towards contraceptive methods utilization. So, as male partner`s educational level increase they will motivate and support their wives to utilize contraceptive methods, in addition, the previous study in Turkey also support husband educational level has a significant association in preference and utilization of modern contraceptive methods (29). 

The current study revealed that women who have debated about modern contraceptive methods with their husbands were 6.09 times more likely to utilize modern contraceptive methods than those who did not. This result was similar to a study done in Gondar town and Ethiopia (17, 30). This might be due to the discussion that can result in effective decision on FP method choice and utilization and the presence of discussion in the rural area showed that there might be good awareness about the FP methods. 

The finding of this study revealed that marital status of women had significantly associated with current contraceptive methods utilization. Married women were 5.88 times more likely to utilize modern contraceptives than unmarried (singled, widowed, and divorced). This finding was in concord with a study done in Gondar town and the study conducted in Tanzania (17, 19). The result indicates the importance of couple inspiration through education and male participation in reproductive health issues including fertility and contraception. So counseling and giving information about FP should inspire joint discussion of fertility issues among couples. However, this finding was not compatible with the finding of the study in Mali (27). The possible reason might be different socio-demographic character and the difference in status of husbands’ habit in two countries. 

Those women who had information about modern contraceptives were 2.63 times more likely to utilize contraceptive methods than those who had not. This finding agrees with a study conducted in Osun state, Nigeria (18). This might be due to Information is the key tool to address FP services in all setting. As the informed people increase, there is a high initiation of utilization and it also used for decision making towards contraceptive methods utilization. 

**Limitation**

Monthly income might be one of the causes that can affect utilization of contraceptives methods but in this study, the assessment of their revenues throughout data collection were not precise so that it was not reflected during the analysis. It also shares the drawbacks of cross-sectional nature of the study. 

## Conclusion

The proportion of modern contraceptive methods utilization among women at the reproductive age group was found to be low. Women`s age, women who have educated husband, marital status and spousal announcement about family planning issues were factors significantly associated with modern contraceptive methods utilization. The policy maker and district health care provider are recommended to increase awareness on modern contraceptive methods, practices referral system to nearby hospital for permanent and long term modern FP services, health education provision to change traditional attitudes towards children that was considered as a benefit for the family, increase counselling about modern contraceptive methods and empowering women on method choice through spousal discussion. 
